# Pentraxin-3 Is a Strong Biomarker of Sepsis Severity Identification and Predictor of 90-Day Mortality in Intensive Care Units via Sepsis 3.0 Definitions

**DOI:** 10.3390/diagnostics11101906

**Published:** 2021-10-15

**Authors:** Huan Chen, Tao Li, Shanshan Yan, Meidong Liu, Ke Liu, Huali Zhang, Min Gao, Xianzhong Xiao

**Affiliations:** 1 Postdoctoral Research Station of Clinical Medicine & Department of Hematology, The Third Xiangya Hospital, Central South University, Changsha 410013, China; chenhuan-1990@foxmail.com; 2 Sepsis Translational Medicine Key Laboratory of Hunan Province, Department of Pathophysiology, School of Basic Medicine Science, Central South University, Changsha 410078, China; liumd2005@126.com (M.L.); lkbingsheng@126.com (K.L.); xiaoxianzhong@csu.edu.cn (X.X.); 3 Department of Pathophysiology, Medical College of Jiaying University, Meizhou 514031, China; lt2016@csu.edu.cn; 4 Department of Critical Care Medicine, The Third Xiangya Hospital, Central South University, Changsha 410013, China; yanshanshan0913@126.com

**Keywords:** pentraxin-3, sepsis, septic shock, biomarker, prognosis, intensive care units

## Abstract

Background: Sepsis is the leading cause of mortality in intensive care units (ICUs). However, early diagnosis and prognosis of sepsis and septic shock are still a great challenge. Pentraxin-3 (PTX3) was shown to be associated with the severity and outcome of sepsis and septic shock. This study was carried out to investigate the diagnostic and prognostic value of PTX3 in patients with sepsis and septic shock based on Sepsis 3.0 definitions. Methods: In this single-center prospective observational study, all patients’ serum was collected for biomarker measurements within 24 h after admission. Logistic and Cox regression analyses were used to identify the potential biomarkers of diagnosis, severity stratification, and prediction. Results: Serum levels of PTX3 were significantly increased on the first day of ICU admission, while septic shock patients had highest PTX3 levels than other groups. A combination between PTX3 and procalcitonin (PCT) could better discriminate sepsis and septic shock, and PTX3 was an independent predictor of mortality in sepsis and septic shock patients. Conclusion: PTX3 may be a robust biomarker to classify the disease severity and predict the 90-day mortality of sepsis and septic shock based on the latest Sepsis 3.0 definitions.

## 1. Introduction

Sepsis is a life-threatening organ dysfunction caused by a dysregulated host response to infection, the leading cause of death in intensive care units (ICUs) [1,2]. Despite recent progress in the diagnosis and intervention, the mortality of sepsis and septic shock remains as high as about 30% in hospitalized patients [3,4]. Considering the complexity of pathogenesis, variability of clinical phenotypes, and high mortality of sepsis and septic shock, a more accurate recognition and risk stratification of these clinical syndromes would allow precise therapeutic strategies and improve patients’ quality of life [5]. In these circumstances, serum biomarkers may improve early diagnosis and severity classification and implement targeted management and appropriate therapies.

Since the latest Sepsis 3.0 definitions focus more on organ dysfunction, traditional biomarkers, such as procalcitonin (PCT) and C-reaction protein (CRP), are conflicting in terms of precise diagnosis and stratification [6,7,8,9]. Therefore, better biomarkers for the diagnosis and prognosis of sepsis are urgently needed.

Pentraxin-3 (PTX3), as an essential component of innate immunity, is upregulated in various infectious diseases [10,11,12]. It could be released by several cell types, such as neutrophils, monocytes, and vascular endothelial cells in response to diverse stimuli [10,13]. It is involved in the defense of specific pathogens and regulation of inflammatory responses [14,15]. Recently, studies suggest that PTX3, along with other biomarkers (such as Interleukin-6 (IL-6), PCT, troponin T, monocyte chemoattractant protein 1 (MCP), proadrenomedullin (proADM), or angiopoietin (Ang) 1/2)) are closely relevant to the severity of patients with sepsis and septic shock and outcome prediction of sepsis and may be a potential biomarker of disease stratification [16,17,18,19,20,21,22].

However, the information about the distribution of PTX3 in all patients admitted to ICUs (including other critically ill patients) was limited, and less evidence was provided about the diagnostic and prognostic value of PTX3 in combination with PCT, especially concerning the latest Sepsis 3.0 definitions. Thus, the present study aimed to investigate the diagnostic and prognostic values of circulating PTX3 among sepsis patients with or without septic shock and critically ill patients at the first 24 h of admission following Sepsis 3.0 definitions.

## 2. Materials and Methods

### 2.1. Study Design and Ethics Statement

This single-center prospective observational study was conducted from April 2018 to February 2019 at the Third Xiangya Hospital of Central South University (No: 2018-S178). The protocol was approved by the Ethics Committee of the Third Xiangya Hospital of Central South University. Written informed consent was obtained from each participant or their legal representatives. All procedures were carried out in accordance with the Declaration of Helsinki, and all experiments were performed by relevant guidelines and institutional regulations.

One hundred and ninety-five patients diagnosed with sepsis and septic shock were recruited from the Third Xiangya Hospital ICU. Thirty-three critically ill patients without sepsis were enrolled and served as the control group. Besides, twenty-four age- and sex-matched healthy volunteers were included as healthy controls when detecting the baseline confirmation of the candidate biomarker.

### 2.2. Definitions

The diagnostic criteria to identify ICU patients with sepsis were defined according to the Sepsis 3.0 definition, which refers to life-threatening organ dysfunction caused by a dysregulated host response to infection, and organ dysfunction can be identified as an rapid change in sequential organ failure assessment (SOFA) score in response to infection [1]. In addition, infection can be identified in the patient who shows positive pathogen culture results as well as positive clinical manifestation or radiographic evidence [23,24].

Criteria of septic shock were defined as follows [1]: (1) persisting hypotension, which needs vasopressors (e.g., dopamine, norepinephrine, epinephrine, vasopressin, phenylephrine, etc.) to maintain mean arterial pressure (MAP) ≥65 mmHg; and (2) serum lactate >2 mmol/L (>18 mg/dL).

SOFA score is currently used to clinically characterize a septic patient, which may be an appropriate tool criteria for auxiliary diagnosis of sepsis, while Acute Physiology and Chronic Health Evaluation II (APACHE II) score is a common classification system of disease severity, which can evaluate the severity by quantizing the degree of multiple physiological parameters [1,2,23]. Considering the close association between coagulation dysfunction and death of sepsis, we also included disseminated intravascular coagulation (DIC) score to assess coagulation disorders in participants [25].

The exclusion criteria included: (1) aged <18 years; (2) the presence of congenital or secondary immunodeficiency disease, bone marrow transplant, or immunosuppressive treatment; (3) rejection of sampling; (4) incomplete data; and (5) ICU stay time <24 h. 

### 2.3. Sampling and Clinical Data Collection

After admission to ICU, patients’ data were collected, containing demographic characteristics (age and gender); the site of primary infection; and underlying diseases, including hypertension, diabetes, chronic diseases, dyslipidemia, neoplasm, etc. [26,27]. SOFA score and APACHE II score were documented within 24 h after ICU stay to determine the disease severity. Besides, results of laboratory tests (such as PCT, CRP, creatinine, platelet count, blood pressure, etc.) were collected within 24 h after ICU admission (or study enrollment) and during follow-up, as was clinical outcome during the ICU stay. In addition, serum samples were obtained within 24 h from 228 enrolled patients after ICU admission (or study enrollment, whichever came first) and 24 healthy volunteers and were frozen at −80 °C until analysis.

### 2.4. Enzyme-Linked Immunosorbent Assay (ELISA)

According to the manufacturers’ instructions, serum levels of PTX3 were measured in participants using DuoSet ELISA detection kits (R & D systems, Minneapolis, MN, USA). 

### 2.5. Statistical Analysis

IBM SPSS 19.0 (SPSS Inc., Chicago, IL, USA) and GraphPad 7.0 (GraphPad Inc., San Diego, CA, USA) were used for statistical analysis and figure preparation. Since all variables were not a strictly normal distribution, continuous data were presented as the median and interquartile range (IQR) and compared by nonparametric tests (the Mann–Whitney U test for two groups; the Kruskal–Wallis test for multiple groups, and the Bonferroni test for two comparisons within groups). Categorical data were presented as number (*n*) and percentage (*%*) and analyzed by the Chi-square test or the Fisher’s exact test. Correlations between PTX3 and traditional indicators were assessed using Spearman’s rank correlation analysis. Univariate and multivariate logistic regression analysis was applied to select diagnostic biomarkers, the receiver operating characteristic (ROC) curves and area under the curve (AUC) were drawn for sensitivity and specificity of diagnostic models, and the leave-one-out cross-validation (LOOCV) test was used to estimate the stability and accuracy of these models [28]. The optimal cut-off value was determined according to the maximum sum of sensitivity and specificity. Survival analysis was performed using the Kaplan–Meier estimator and compared with the log-rank test between high and low levels of candidate biomarkers (dichotomized by cut-off values) from the time of ICU admission (or study enrollment) until death from any cases at day 90. Patients alive at the endpoint of follow-up were censored. Then, 90-day mortality was predicted by univariate and multivariate Cox regression analysis and reported as hazard ratio (HR) and 95% confidence interval (CI). A two-tailed *p*-value of <0.05 was taken as a cut-off for statistical significance, and analysis was adjusted by gender and age. 

## 3. Results

### 3.1. Clinical Characteristics in Sepsis, Septic Shock, and Non-Sepsis ICU Patients

**Figure 1:**
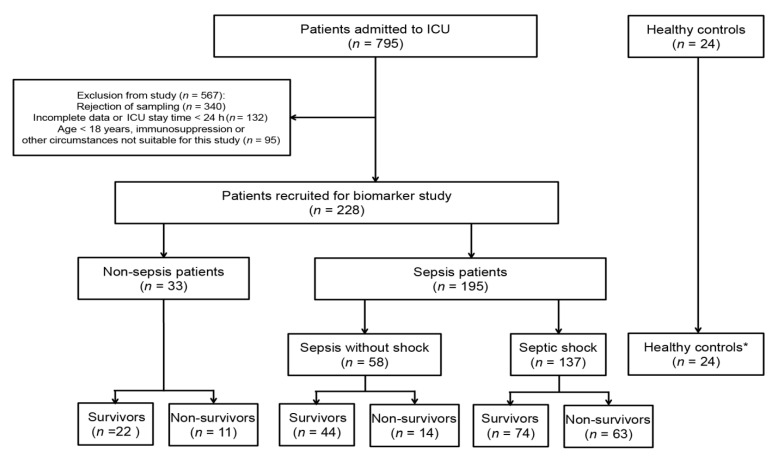
Flow chart of the study population. * Healthy controls were not used for analysis.

Infection was the most common reason for ICU admission and mortality, and sepsis constituted more than half of the cases. Of 795 patients admitted to ICU, 340 patients were excluded for rejection of consents, 132 patients were excluded due to the incomplete data or less than 24 h ICU stay time, and 95 patients met other exclusion. Subsequently, 33 patients who did not suffer from infection or total SOFA score <2 were selected as a group of critical illness without sepsis. Finally, 195 ICU sepsis patients, including sepsis without shock (*n* = 58) and septic shock (*n* = 137), as well as 33 critically ill patients without sepsis, were enrolled in the present study (Figure 1).

All participants’ demographic and clinical characteristics (including 90-day mortality) are listed in Table 1. Notably, unlike the healthy controls, although significant differences were seen between non-sepsis patients and total sepsis patients, there was a barely significant difference in most clinical characteristics between sepsis patients without shock and non-sepsis patients except for ICU stay time and infection or not (Table 1). However, patients with septic shock showed worse features compared with the other two groups of patients, including higher serum levels of PCT and lactate, lower platelet count, higher SOFA score, DIC score, and APACHE II score, as well as a higher risk of death (septic shock: 46%, vs. sepsis without shock: 24% and non-sepsis: 33%) (Table 1).

### 3.2. Distribution of Serum PTX3

**Figure 2:**
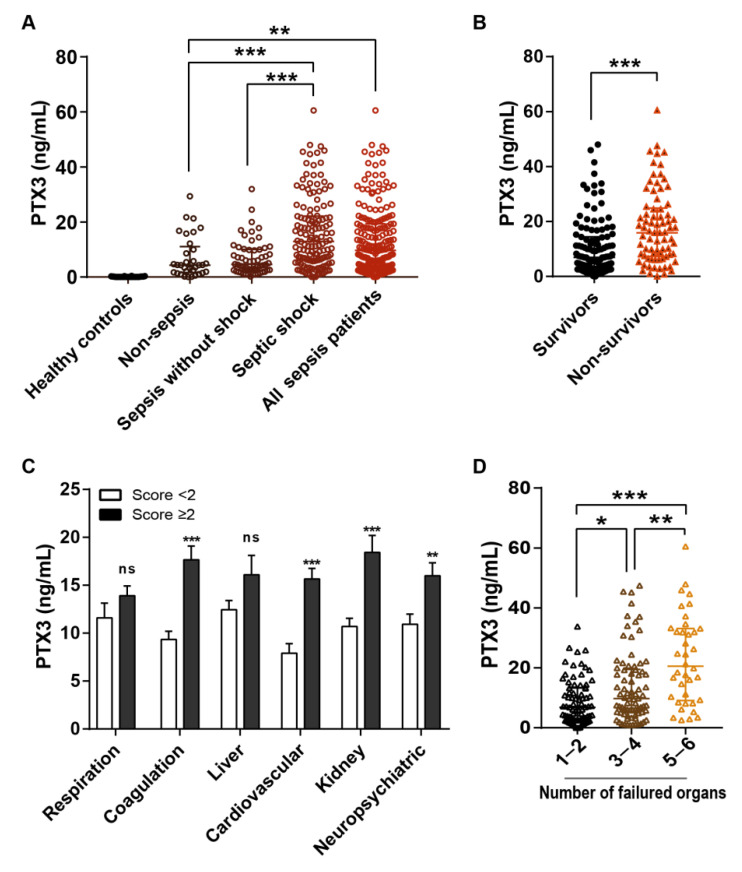
Pentraxin-3(PTX3) levels and demographics. These panels show the existing differences in serum levels of PTX3 in ICU patients by selected demographics. PTX3 levels were higher in sepsis and septic shock patients (**A**), in non-survivors with 90-day (**B**), in patients with sub-SOFA score > 2 (**C**), and in patients with more failure organs (**D**). Concentrations of PTX3 were measured by ELISAs. Data are presented as the median and interquartile range (Mann–Whitney U test or Kruskal–Wallis test, respectively). * *p* < 0.05, ** *p* < 0.01, *** *p* < 0.001, Here “ns” means “ not significant”.

The concentrations of PTX3 in septic shock patients within 24 h after admission (or study enrollment) were the highest in the overall cohort. The levels of PTX3 were raised in most ICU patients compared to those in healthy volunteers, as shown in Figure 2A. In comparison among sub-groups, a significant increase in PTX3 was seen in septic shock patients, whereas similar distribution was seen between non-sepsis patients and sepsis without shock patients (Figure 2A). Besides, the serum concentrations of PTX3 were dramatically raised in non-survivors compared to that in survivors (*p* < 0.001) in patients diagnosed with sepsis (Figure 2B). Moreover, the levels of PTX3 were remarkably higher in patients with severe organ dysfunctions, characterized by whose SOFA score of sub-groups, including coagulation, cardiovascular, kidney, and neuropsychiatric, were higher than 2 (Figure 2C), and raised along with the increased numbers of failure organs (Figure 2D), which suggested that PTX3 appeared to be linked with the degree of the severity of sepsis and multiple organ dysfunction syndrome (MODS).

### 3.3. Correlations of PTX3 with Other Disease Severity Associated Variables

**Figure 3:**
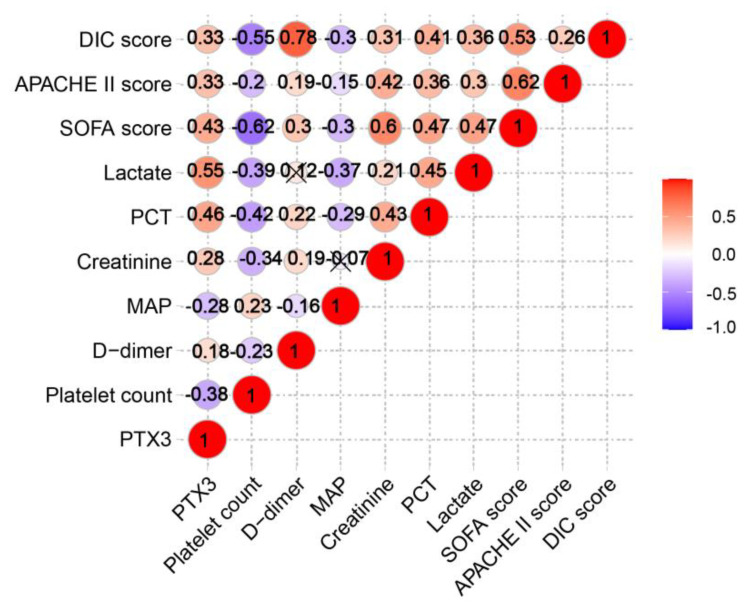
Correlations of PTX3 with traditional severity parameters in ICU patients with sepsis and septic shock. The significance of correlations is shown in the upper correlation heatmap.

Since PTX3 seemed to be associated with the severity of sepsis, Spearman bivariate correlation analysis was evaluated between PTX3 and the following clinical indicators that reflected organ failures and disease severity. As shown in Figure 3, higher PTX3 concentrations were strongly associated with higher serum lactate (*r* = 0.55, *p* < 0.001), higher serum PCT (*r* = 0.46, *p* < 0.001), higher SOFA score (*r* = 0.43, *p* < 0.001), higher DIC score (*r* = 0.33, *p* < 0.001), higher APACHE II score (*r* = 0.33, *p* < 0.001), higher serum creatinine (*r* = 0.28, *p* < 0.001), higher d-dimer (*r* = 0.18, *p* = 0.01), as well as with lower platelet count (*r* = −0.38, *p* < 0.001) and lower mean arterial pressure (MAP) (*r* = −0.28, *p* < 0.001).

### 3.4. Diagnostic Value of PTX3 in Discriminating Sepsis and Septic Shock according to the Sepsis 3.0 Definitions

To investigate whether any single or combined biomarkers could be used to discriminate sepsis and septic shock, we included PTX3 and other positive biomarkers, such as PCT, lactate, and platelet count, in the univariate and multivariate logistic regression analysis.

**Figure 4:**
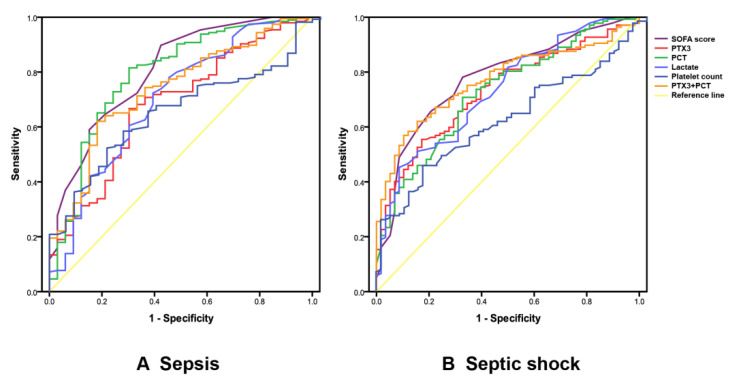
Receiver-operating characteristic (ROC) curves for diagnosing sepsis (**A**) and septic shock (**B**) by pentraxin-3 (PTX3), procalcitonin (PCT), lactate, and platelet count.

Univariate analysis demonstrated that PTX3, PCT, lactate, and platelet count were significantly associated with the diagnosis and severity of sepsis. PTX3 showed relatively better performance (accuracy and stability) for the diagnosis and stratification of sepsis, with the cut-off value of 11.12 ng/mL (*p* = 0.001, *p* < 0.001, respectively; Table 2 and Figure 4), although PCT showed larger AUC-ROC instead of better stability.

We further assessed whether the combinations of biomarkers could increase the accuracy of discrimination. Using a stepwise multivariate analysis, we screened out a combination of PTX3 and PCT to increase the specificity of diagnosis and severity stratification, correctly identifying 68.2% sepsis patients and correctly classifying 69.3% septic shock patients (Table 2 and Figure 4).

### 3.5. Prognostic Value of PTX3

**Figure 5:**
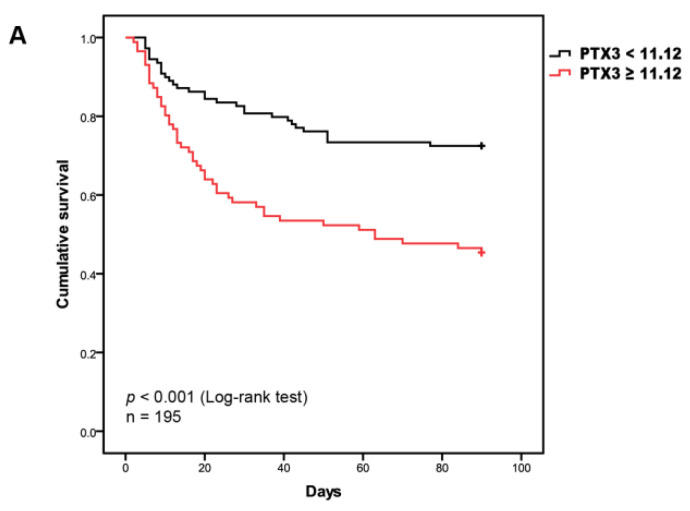

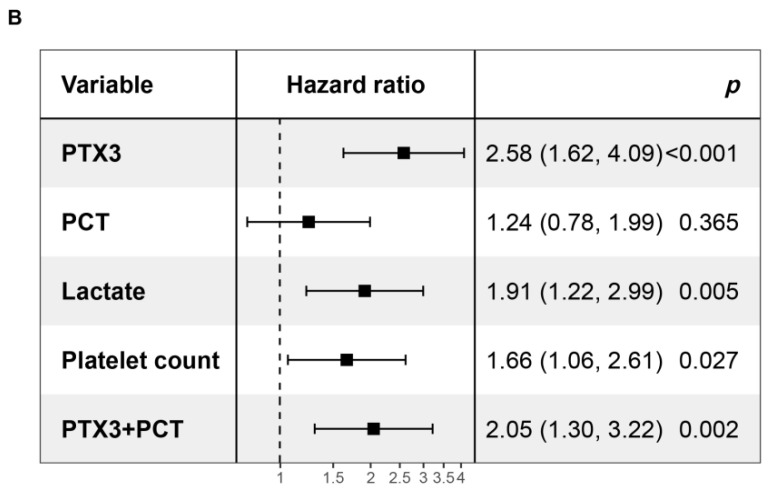
Survival analysis for sepsis patients without or with shock. (**A**) *Kaplan–Meier* survival curves for patients between high and low levels of pentraxin-3 (PTX3), the dashed lines represent those with PTX3 levels above the cut-off value (11.12 ng/mL). (**B**) Forest plot of adjusted hazard ratios for septic shock patients relative to sepsis patients without shock with levels of biomarkers below cut-off value by univariate and multivariate *Cox* regression analysis. Hazard ratios are adjusted for age and gender.

To estimate the prognostic value of these potential biomarkers, we created Kaplan–Mayer survival curves for the incidence of sepsis mortality during 90 days follow-up, comparing under and over cut-off values for the candidate indicators including PTX3, PCT, lactate, and platelet count. Results from Kaplan–Meier curves revealed that higher levels of PTX3 (≥11.12 ng/mL) were statistically associated with lower survival rate within 90 days (*p* < 0.001, log-rank test; Figure 5A), paralleled with lactate (cutoff value: 3.9 mmol/L; *p* = 0.004, log-rank test) and platelet count (cutoff value: 87 × 10^12^/L; *p* = 0.004, log-rank test) (see Figure S1).

However, univariate and multivariate Cox regression analysis revealed that PTX3 alone might be a better risk factor for 90-day mortality (hazard ratio = 2.58; 95% CI: 1.62–4.09; *p* < 0.001), although lactate and platelet count were also associated with 90-day mortality in sepsis patients, which was displayed by forest plot (Figure 5B).

## 4. Discussion

Sepsis has been universally known as a major cause of morbidity in the ICU. Its mortality is intimately associated with the disease severity of sepsis and the degrees of organ dysfunctions [2]. In the present study, the mortality rate of patients with septic shock was twice that of sepsis patients without shock, consistent with prior studies [3,29]. Thus, early and precise septic shock identification and accurate survival prediction are crucial to clinical risk stratification and advanced management. In the current study, critically ill patients admitted to ICU were enrolled as control groups to analyze the diagnosis and prognosis of sepsis and septic shock to obtain more reliable results in the real world.

In the present study, we revealed that (1) the PTX3 concentrations were remarkably raised within 24 h after admission to ICU, compared with healthy volunteers, particularly in patients with septic shock; (2) higher PTX3 were related with higher mortality; and (3) the PTX3 levels were closely associated with the number and degree of organ dysfunctions, as indicated by the correlations among PTX3 and the SOFA scores for all sub-groups as well as severity parameters. Most of these findings were in line with previous studies [30,31,32,33,34], for instance, higher levels of circulating PTX3 in non-survivors in patients with septic shock compared to survivors [27], and higher PTX3 were correlated with coagulation dysfunction [30], cardiovascular failure [32,34] or renal failure [33] in severe sepsis/acute coronary syndrome/chronic kidney disease. In addition, severity parameters, such as lactate, platelet count, d-dimer, creatinine, MAP, DIC score, and APACHE II score, was closely correlated with PTX3, indicating consistent changes with corresponding organ dysfunctions and reflecting the role of PTX3 in endothelial damage and vascular dysfunction [13,35,36]. However, PTX3 levels were not significantly different between the non-sepsis group and sepsis without shock group in patients admitted to the ICU, similar to other clinical indicators, including PCT, CRP, lactate, creatinine, d-dimer, APACHE II score, and SOFA score, which may partly interpret the difficulty in the diagnosis of sepsis in the ICUs. Simultaneously, these findings may explain the close relationship between PTX3 and the number and degree of organ dysfunctions, which suggest that elevated circulating PTX3 were involved in the development of organ failures, not related to the causes of hospitalization or infection to a certain extent.

In terms of investigating the diagnostic value of early detection of PTX3, we found that PTX3, PCT, lactate, and platelet count could distinguish septic shock patients from other patients admitted to the ICU with the best performance. Unlike other research, the controlled population in the present study is critically ill patients in ICUs instead of healthy volunteers, emergency patients, or in-hospital patients [27,37,38]. Since critically ill patients in ICUs are more or less suffered from organ injury, most of the clinical indicators between sepsis patients and non-sepsis patients in ICUs are remarkably similar. It is hard to distinguish between them. Compared with other indicators, PTX3 showed more specificity in differentiating septic shock patients from non-shock sepsis patients (cut-off value: 11.12 ng/mL), higher AUC-ROC values, and more stable and accurate (using LOOCV test). After multivariate analysis, we found that PTX3 combined with PCT could improve the diagnostic value to distinguish between septic shock patients and other critically ill patients, paying more attention to managing patients at high risk in time. 

Furthermore, in terms of the prognostic value, PTX3, lactate, and platelet count were closely linked with 90-day mortality. Multivariate analysis revealed that PTX3 alone was a better risk factor for mortality, whether in sepsis and septic shock patients. Our study suggests that the PTX3 is a reliable predictor for poor outcome and 90-day mortality and can improve patient management and therapy based on the risk stratification in the population of ICU patients. 

Considering sepsis’s complexity and diagnostic difficulties, the current predictive scoring systems still have limitations, including poor generalizability, possible bias of diagnosis window, and fast aggravation over time. They also seem to be used more in the field of research. Given this, although PTX3 did not perform the best in distinguishing sepsis patients from other critically ill patients, it should not be excluded for the application of auxiliary diagnosis and prediction in routine clinical settings.

Our results are consistent with previous studies that confirmed PTX3 as a prognostic biomarker for sepsis [16,17,18,19,20,21]. Higher levels of PTX3 were seen in non-survivors compared to survivors and were linked with the severity of sepsis. A large observational study demonstrated that PTX3 levels higher than 6.4 ng/mL of non-hospitalized individuals were remarkably linked with higher mortality, independently of hospitalization causes [39]. Thus, PTX3 may be an independent predictor of bad outcomes in sepsis. Notably, in most of these studies, healthy volunteers or relatively mild patients were enrolled as controls. Fewer studies investigated the distribution of PTX3 and its diagnostic and prognostic value among patients with sepsis or other critical illness. Therefore, our findings provided more evidence for the potential value of PTX3 in sepsis diagnosis and prognosis, that is, early detection of PTX3 in patients within 24 h admitted to ICU may be helpful to identify septic patients with possible shock or high death risks, which can prompt physicians to strengthen patient management and improve patient outcomes. To sum up, PTX3 may be a potential biomarker in the diagnosis and prognosis of septic shock patients in clinical practice.

There are several limitations to this study. First, the present study was a single-center prospective study performed at a tertiary referral hospital. The group of patients included in this study was relatively small, and further prospective multi-center study is needed to validate the results. Second, some patients transferred to our hospital have been treated with prior therapies, such as antibiotics, vasopressors, or fluid resuscitation.

## 5. Conclusions

PTX3 was increased in serum of critically ill patients and highly upregulated in septic shock patients. Meanwhile, the expression of PTX3 was strongly correlated with organ dysfunction and the severity of sepsis. Additionally, our study suggests that PTX3 combined with PCT may be early biomarkers to discriminate sepsis and septic shock with other critically ill patients according to the Sepsis 3.0 definitions, and PTX3 may play an influential role in predicting 90-day mortality of sepsis for the guidance of advanced therapy and management.

## Figures and Tables

**Table 1:** Demographic, clinical, and laboratory characteristics of patients enrolled in the study.

Variables	Non-Sepsis Patients (*n* = 33)	Sepsis Patients			*p* Value (Non-Sepsis vs. Total Sepsis)	Healthy Controls (*n* = 24)
		Total Sepsis Patients (*n* = 195)	Sepsis without Shock Patients (*n* = 58)	Septic Shock Patients (*n* = 137)		
Age (y), M (IQR)	52 (36–72)	60 (47–70)	58 (47–70)	62 (48–70)	0.262	47 (40–50)
Male sex, *n* (%)	16 (49)	120 (62)	35 (60)	85 (62)	0.157	14 (58)
Numbers of infection site, M (IQR)	1 (0–1)	2 (1–2) ^&&&^	2 (1–2) ***	2 (1–2) *** ^#^	<0.001	—
**Infection Site**						
Pulmonary, *n* (%)	21 (64)	140 (72)	43 (74)	97 (71)	0.341	—
Urinary, *n* (%)	1 (3)	41 (21) ^&^	11 (19) *	30 (22) *	0.013	—
Intra-abdominal, *n* (%)	4 (12)	83 (43) ^&&^	25 (43) **	58 (42) **	0.001	—
Blood-borne, *n* (%)	0 (0)	54 (28) ^&&&^	8(14) ***	46 (34) ***	<0.001	—
Skin and soft tissue, *n* (%)	3 (9)	24 (12)	6 (10)	18 (13)	0.775	—
**Underlying Diseases**						
Perioperative period, *n* (%)	13 (39)	107 (55)	33 (57)	74 (54)	0.100	—
Hypertension, *n* (%)	11 (33)	65 (33)	20 (35)	45 (33)	1.000	—
Diabetes, *n* (%)	3 (9)	43 (22)	14 (24)	29 (21)	0.103	—
Chronic lung disease, *n* (%)	4 (12)	21 (11)	7 (12)	14 (10)	0.767	—
Chronic liver disease, *n* (%)	6 (18)	9 (5) ^&&^	2 (4) *	7 (5) *	0.004	—
Chronic kidney disease, *n* (%)	6 (18)	24 (12)	7 (12)	17 (12)	0.356	—
Dyslipidemia, *n* (%)	3 (9)	42 (22)	14 (24)	28 (20)	0.153	—
Neoplasm, *n* (%)	4 (12)	34 (17)	10 (17)	24 (18)	0.615	—
**Infection and Inflammation-Associated Laboratory Markers**						
PCT(ng/mL), M (IQR)	0.94 (0.50–3.71)	12.38 (2.64–43.84) ^&&&^	3.65 (1.08–15.48)	16.00 (4.75–62.39) *** ^###^	<0.001	—
CRP(mg/L), M (IQR)	132.9 (32.8–188.2)	168.3 (71.5–200.0)	89.3 (37.9–200.0)	170.8 (83.2–200.0)	0.099	—
Lactate(mmol/L), M (IQR)	1.8 (1.1–3.1)	3.0 (2.1–5.1) ^&&&^	2.3 (1.4–3.1)	3.6 (2.4–6.4) *** ^###^	<0.001	—
Bilirubin(μmol/L), M (IQR)	13.9 (6.1–21.3)	19.1 (9.6–34.9) ^&&^	16.7 (7.3–24.23)	20.7 (9.8–42.8) **	0.009	—
Creatinine(μmol/L), M (IQR)	75 (64–224)	123 (75–255)	91 (63–181)	137 (88–265) ^#^	0.075	—
WBC count (10^9^/L), M (IQR)	12.15 (9.13–14.01)	11.43 (7.78–17.95)	11.11 (7.41–15.02)	12.40 (7.81–18.25)	0.709	—
Platelet count (10^12^/L), M (IQR)	169 (118–205)	107 (62–183) ^&&^	136 (91–202)	93 (41–173) ** ^#^	0.004	—
D-dimer(mg/L), M (IQR)	3.82 (1.84–8.11)	6.45 (3.64–11.43) ^&&^	5.78 (2.72–9.94)	6.97 (3.87–12.23) **	0.007	—
MAP, M (IQR)	95 (80–108)	78 (67–92) ^&&&^	91 (80–106)	75 (65–85)	<0.001	—
DIC score, M (IQR)	2 (0–3)	3 (2–5) ^&&^	2 (1–4)	4 (2–5) *** ^##^	0.003	—
APACHE II score, M (IQR)	14 (9–20)	19 (13–24) ^&&^	16 (12–20)	20 (15–25) ** ^#^	0.008	—
SOFA score, M(IQR)	4 (3–7)	10 (6–13) ^&&^	6 (5–9)	11 (8–14) *** ^###^	<0.001	—
Mechanical ventilation, *n* (%)	22 (66.7)	158 (81)	42 (72.4)	116 (84.7) ^#^	0.061	—
ICU stay (h), M (IQR)	96 (62–189)	142 (87–281) ^&^	182 (93–253) *	139 (85–284)	0.040	—
90-day mortality, *n* (%)	11 (33)	77 (40)	14 (24)	63 (46) * ^#^	0.502	—

(1) * vs. non-sepsis patients. # vs. sepsis without shock patients. & represents compared between non-sepsis and total sepsis. */#/& *p* < 0.05, **/##/&& *p* < 0.01, ***/###/&&& *p* < 0.001. (2) APACHE II: Acute Physiology and Chronic Health Evaluation II; CRP: C-reaction protein; DIC: disseminated intravascular coagulation; ICUs: intensive care units; M (IQR): median (interquartile range); MAP: mean arterial pressure; PCT: procalcitonin; SOFA: sequential organ failure assessment; WBC: white blood cell.

**Table 2:** Diagnostic value of univariate and multivariate biomarkers for patients with sepsis and septic shock.

Variable	Severity	AUC (95% CI)	Cut-Off Value	Sensitivity	Specificity	*p* Value	True Positive Rate *	True Negative Rate *
Univariate analysis								
PTX3	Sepsis	0.68 (0.58–0.78)	5.84	0.667	0.697	0.001	0.467	0.758
(ng/mL)	Septic shock	0.73 (0.66–0.80)	11.12	0.555	0.828	<0.001	0.526	0.828
PCT	Sepsis	0.79 (0.70–0.88)	1.62	0.815	0.697	<0.001	0.344	0.879
(ng/mL)	Septic shock	0.73 (0.65–0.80)	7.27	0.328	0.672	<0.001	0.409	0.879
Lactate	Sepsis	0.70 (0.60–0.80)	2.3	0.723	0.621	<0.001	0.405	0.848
(mmol/L)	Septic shock	0.73 (0.66–0.81)	3.9	0.453	0.914	<0.001	0.423	0.914
Platelet count	Sepsis	0.66 (0.57–0.74)	132	0.585	0.727	0.004	0.677	0.545
(×10^12^/L)	Septic shock	0.63 (0.55–0.71)	87	0.460	0.828	0.004	0.664	0.431
Multivariate analysis								
PTX3 + PCT	Sepsis	0.74 (0.65–0.82)	85.5	0.621	0.818	<0.001	0.682	0.667
(score)	Septic shock	0.77 (0.70–0.84)	74.8	0.569	0.897	<0.001	0.693	0.724

(1) logistic analysis was adjusted by sex and age. * By Leave-One-Out Cross-Validation. (2) AUC: area under the curve; CI: confidence interval; PCT: procalcitonin; PTX3: pentraxin-3.

## Data Availability

The study data is not available in a public database. However, data can be requested at the corresponding authors.

## Supplementary Materials

The following are available online at https://www.mdpi.com/article/10.3390/diagnostics11101906/s1, Figure S1: Survival analysis for sepsis patients without or with shock.

## Abbreviations

APACHE II: Acute Physiology and Chronic Health Evaluation II; AUC: area under curve; CI: confidence interval; CRP: C-reaction protein; DIC: disseminated intravascular coagulation; HR: hazard ratio; ICUs: intensive care units; IQR: interquartile range; LOOCV: leave-one-out cross validation; MAP: mean arterial pressure; PCT: procalcitonin; proADM: proadrenomedullin; PTX3: pentraxin-3; ROC: receiver operating characteristic; SOFA: sequential organ failure assessment; WBC: white blood cell.
