# Supplementary material for: Pentraxin-3 Is a Strong Biomarker of Sepsis Severity Identification and Predictor of 90-Day Mortality in Intensive Care Units via Sepsis 3.0 Definitions
Source: Diagnostics (Basel). 2021 Oct 15;11(10):1906. doi: 10.3390/diagnostics11101906 (PMC8534382; doi:10.3390/diagnostics11101906)
Supplement: Supplementary file 1 [file diagnostics-11-01906-s001.zip › Manuscript-0906 supplementary meterial.pdf]

diagnostics

Article

Pentraxin-3 Is A Strong Biomarker of Sepsis Severity Identification and Predictor of 90-Day Mortality in Intensive Care Units via Sepsis 3.0 Definitions

Huan Chen [1,2], Tao Li [3], Shanshan Yan [4], Meidong Liu [2], Ke Liu [2], Huali Zhang [2,*], Min Gao [4,*] and Xianzhong Xiao [2]

1 Postdoctoral Research Station of Clinical Medicine & Department of Hematology, The Third Xiangya Hospital, Central South University, Changsha 410013, China; chenhuan-1990@foxmail.com
2 Sepsis Translational Medicine Key Laboratory of Hunan Province, Department of Pathophysiology, School of Basic Medicine Science, Central South University, Changsha 410078, China; liumd2005@126.com (M.L.); lkbingsheng@126.com (K.L.); xiaoxianzhong@csu.edu.cn (X.X.)
3 Department of Pathophysiology, Medical College of Jiaying University, Meizhou 514031, China; lt2016@csu.edu.cn
4 Department of Critical Care Medicine, The Third Xiangya Hospital, Central South University, Changsha 410013, China; yanshanshan0913@126.com
* Correspondence: zhanghuali@csu.edu.cn (H.Z.); gaomin1@csu.edu.cn (M.G.)

Citation: Chen, H.; Li, T.; Yan, S.; Liu, M.; Liu, K.; Zhang, H.; Gao, M.; Xiao, X. Pentraxin-3 Is A Strong Biomarker of Sepsis Severity Identification and Predictor of 90-Day Mortality in Intensive Care Units via Sepsis 3.0 Definitions. *Diagnostics* **2021**, *11*, 1906. https://doi.org/10.3390/diagnostics11101906

Academic Editor: Marcello Ciaccio

Received: 6 September 2021
Accepted: 5 October 2021
Published: 15 October 2021

Publisher's Note: MDPI stays neutral with regard to jurisdictional claims in published maps and institutional affiliations.

Copyright: © 2021 by the authors. Licensee MDPI, Basel, Switzerland. This article is an open access article distributed under the terms and conditions of the Creative Commons Attribution (CC BY) license (https://creativecommons.org/licenses/by/4.0/).

Abstract: Background: Sepsis is the leading cause of mortality in intensive care units (ICUs). However, early diagnosis and prognosis of sepsis and septic shock are still a great challenge. Pentraxin-3 (PTX3) was shown to be associated with the severity and outcome of sepsis and septic shock. This study was to investigate the diagnostic and prognostic value of PTX3 in patients with sepsis and septic shock based on Sepsis 3.0 definitions. Methods: In this single-center prospective observational study, all patients' serum was collected for biomarker measurements within 24 h after admission. Logistic and Cox regression analyses were used to identify the potential biomarkers of diagnosis, severity stratification, and prediction. Results: Serum levels of PTX3 were significantly increased on the first day of ICU admission, while septic shock patients had highest PTX3 levels than other groups. A combination between PTX3 and procalcitonin (PCT) could better discriminate sepsis and septic shock, and PTX3 was an independent predictor of mortality in sepsis and septic shock patients. Conclusion: PTX3 may be a robust biomarker to classify the disease severity and predict the 90-day mortality of sepsis and septic shock based on the latest Sepsis 3.0 definitions.

Keywords: Pentraxin-3; sepsis; septic shock; biomarker; prognosis; intensive care units

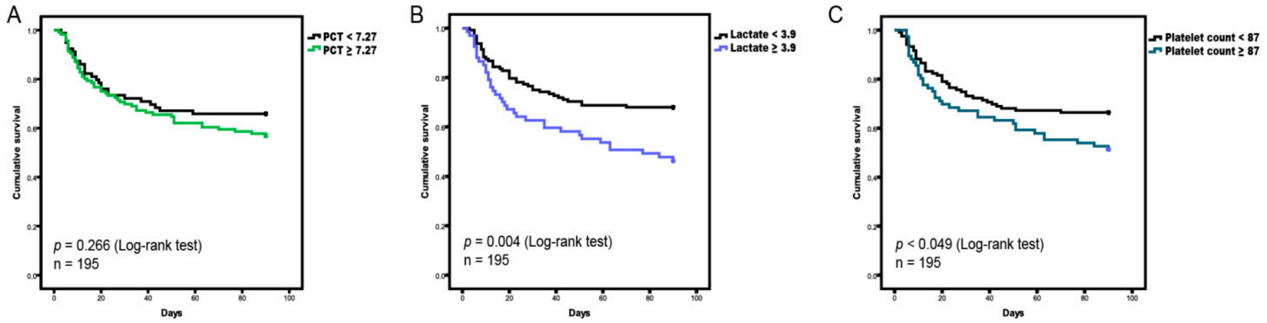

Figure S1. Survival analysis for sepsis patients without or with shock. (A) Kaplan-Meier survival curves for patients between high and low levels of procalcitonin (PCT); the dashed lines represent those with PCT levels above the cutoff value (7.27 ng/mL). (B) Kaplan-Meier survival curves for patients between high and low levels of lactate; the dashed lines represent those with lactate levels above the cutoff value (3.9 mmol/L). (B) Kaplan-Meier survival curves for patients between high and low levels of platelet count; the dashed lines represent those with platelet count levels above the cutoff value (87×10^{12}/L).
