# Supplementary figures and images for: Pentraxin-3 Is a Strong Biomarker of Sepsis Severity Identification and Predictor of 90-Day Mortality in Intensive Care Units via Sepsis 3.0 Definitions
Source: Diagnostics (Basel). 2021 Oct 15;11(10):1906. doi: 10.3390/diagnostics11101906 (PMC8534382; doi:10.3390/diagnostics11101906)

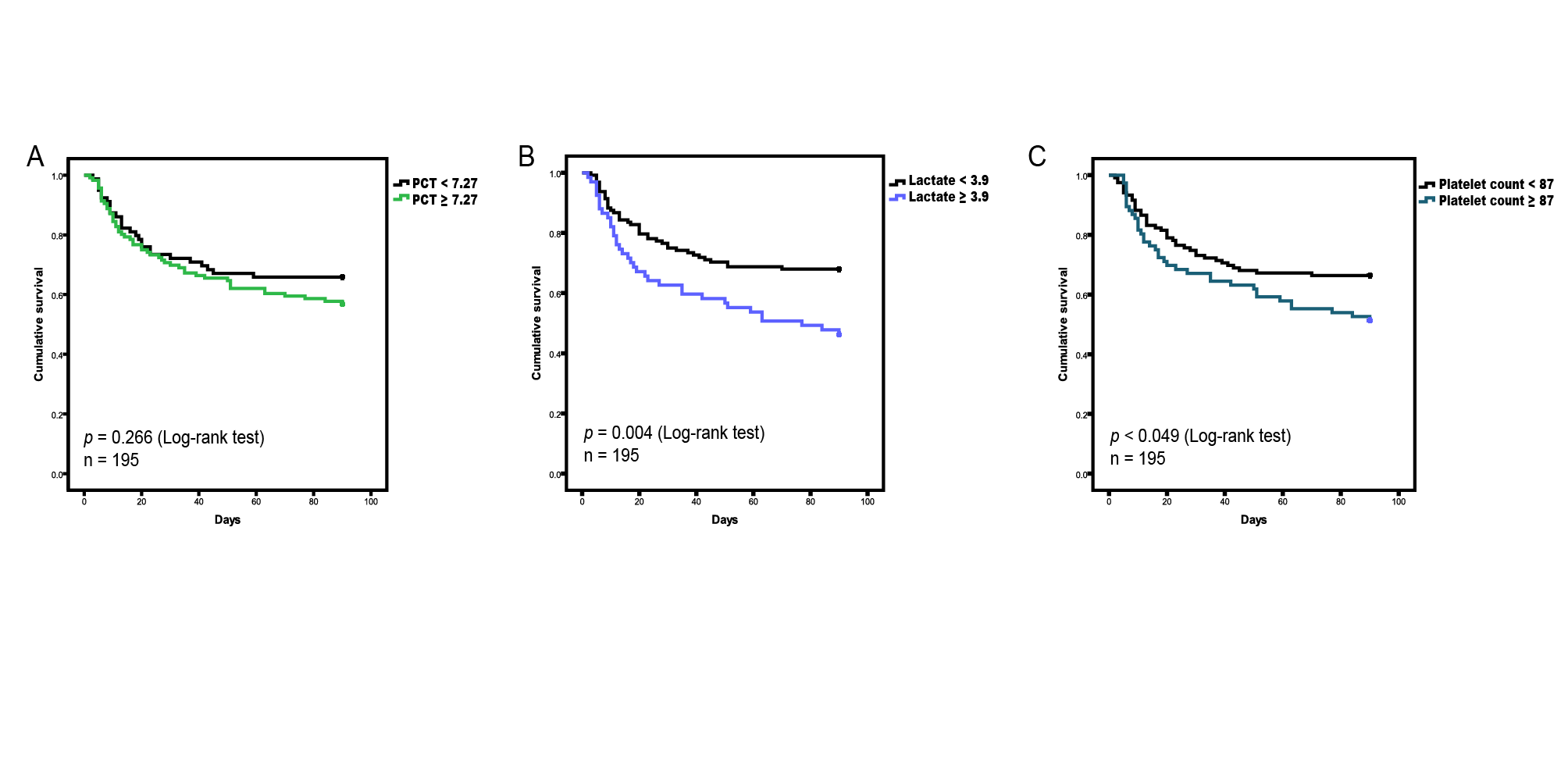

Supplement: Supplementary file 1 [file diagnostics-11-01906-s001.zip › Supplymentary figure S1-1.tif]
